# Appropriate inclusion of interactions was needed to avoid bias in multiple imputation

**DOI:** 10.1016/j.jclinepi.2016.07.004

**Published:** 2016-12

**Authors:** Kate Tilling, Elizabeth J. Williamson, Michael Spratt, Jonathan A.C. Sterne, James R. Carpenter

**Affiliations:** a School of Social and Community Medicine, University of Bristol, Canynge Hall, 39 Whatley Road, Bristol, BS8 2PS, UK; b Department of Medical Statistics, London School of Hygiene and Tropical Medicine, University of London, Keppel Street, London WC1E 7HT, UK; c Farr Institute of Health Informatics, London University College London, 222 Euston Road, London NW1 2DA, UK; d MRC Clinical Trials Unit, University College London, Aviation House 125 Kingsway, London WC2B 6NH, UK

**Keywords:** Multiple imputation, Missing data, Interaction, Complete case analysis, Bias, Simulation

## Abstract

**Objective:**

Missing data are a pervasive problem, often leading to bias in complete records analysis (CRA). Multiple imputation (MI) via chained equations is one solution, but its use in the presence of interactions is not straightforward.

**Study Design and Setting:**

We simulated data with outcome *Y* dependent on binary explanatory variables *X* and *Z* and their interaction XZ. Six scenarios were simulated (*Y* continuous and binary, each with no interaction, a weak and a strong interaction), under five missing data mechanisms. We use directed acyclic graphs to identify when CRA and MI would each be unbiased. We evaluate the performance of CRA, MI without interactions, MI including all interactions, and stratified imputation. We also illustrated these methods using a simple example from the National Child Development Study (NCDS).

**Results:**

MI excluding interactions is invalid and resulted in biased estimates and low coverage. When XZ was zero, MI excluding interactions gave unbiased estimates but overcoverage. MI including interactions and stratified MI gave equivalent, valid inference in all cases. In the NCDS example, MI excluding interactions incorrectly concluded there was no evidence for an important interaction.

**Conclusions:**

Epidemiologists carrying out MI should ensure that their imputation model(s) are compatible with their analysis model.

**What is new?**

**What is known:**
• Passive imputation (or “impute then transform”) for interaction terms can create bias.
• Improving the passive approach relies on correct specification of the imputation models, which is hard to do.

**What is new from our paper:**
• When the interaction term is between two binary variables, it is relatively easy to correctly specify all the imputation models, thus achieving unbiased estimates.
• When one of the variables involved in the interaction is both fully observed and categorical, the data can be split into strata defined by this variable, and imputation (without interaction) carried out separately within each stratum.
• Alternatively, a single imputation model with appropriate interactions has the potential to be more efficient, and is the only option for valid inference when the interaction involves continuous variables.
• Even when the interaction terms are in truth zero, not including them in the imputation model results in overcoverage of confidence intervals.

## 1 Introduction

Missing data are a pervasive problem for most medical and epidemiological studies, with complete records analysis (CRA) in the presence of missing data often leading to bias [1]. One approach is multiple imputation (MI) [2], [3], where multiple plausible values for each missing value are imputed, and the analysis performed on each of the multiple imputed data sets and combined using the Rubin's rules [4]. With the increasing popularity of MI among epidemiologists has come awareness of potential pitfalls [2], many of which center around the need for compatibility between the imputation and analysis models. However, awareness of what this means in practice and the implications of having incompatible imputation and analysis models are not widely known.

If data are missing at random (MAR, [5]) (i.e., the probability of being a complete record depends only on observed data, not on the unobserved (missing) data), appropriate MI has the potential to correct bias and recover information. MI may still be used if data are missing not at random [5] (i.e., the probability of being a complete record depends on the unobserved data), but in this case, additional information needs to be provided by the analyst [6], and this is beyond the scope of this article.

To be compatible with the analysis model, the imputation model should include all the variables in the analysis model (including the response variable) as well as auxiliary variables related to the variables being imputed [7], [8], [9], [10]. When the analysis model includes interactions, the imputation model(s) need to be constructed so as to include all the implied interactions. Our aims are (1) to show how to correctly construct the imputation model to be compatible with interactions in the analysis model and (2) to explore the practical consequences of misspecification using a simulation study and a simple application to real data from the National Child Development Study (NCDS).

## 2 Imputation when the analysis model contains interactions

In this article, we focus on the situation where the analysis model of interest is a regression (linear, logistic) of a response variable *Y* on binary covariates *X*, *Z* and their interaction.

Under the full conditional specification (FCS) algorithm for MI (also known as multiple imputation with chained equations) [11], [12], [13], each partially observed variable is imputed in turn, conditional on the observed data and imputed values of all the other variables, and this process cycled to convergence. For our three-variable example, where each of the response variable *Y* and binary covariates *X* and *Z* may be partially observed, one cycle of FCS imputation consists of

1. regression of observed *X* on (a function of) *Y* and *Z*, where missing values in *Y* and *Z* are replaced by their current imputed values; followed by imputation of missing *X*s
2. regression of observed *Z* on (a function of) *X* and *Y*, where missing values in *X* and *Y* are replaced by their current imputed values; followed by imputation of missing *Z*s
3. regression of observed *Y* on (a function of) *X* and *Z*, where missing values in *X* and *Z* are replaced by their current imputed values; followed by imputation of missing *Y*s

If any of *X*, *Z*, or *Y* is fully observed, then imputation of that variable is not necessary, and so fewer than three imputation models may be needed.

In most statistical software, the default implementation of this approach imputes the partially missing variable (in steps 1–3 mentioned previously) via a regression model on main effects of the other variables only. Interactions or nonlinear relationships within the imputation models must be explicitly specified.

Our analysis model—the regression of *Y* on *X*, *Z* and their interaction, XZ—implies that the relationship between *Y* and *X* varies by *Z* and that the relationship between *Y* and *Z* varies by *X*. Thus, to impute compatibly with this analysis model, we need our imputation models to be

1. regression of *X* on *Y*, *Z*, YZ;
2. regression of *Z* on *X*, *Y*, XY;
3. regression of *Y* on *X*, *Z*, XZ (the analysis model).

Thus, including the interaction between *X* and *Z* in the imputation model for the response variable *Y* is not sufficient to ensure compatibility between imputation and analysis models.

Alternatively, where one of the three variables is fully observed and is categorical, all necessary interactions can be incorporated into the imputation models by stratifying on the fully observed variable and imputing separately within these subsets. For example, if binary *X* were fully observed, we could stratify the data by *X* and then impute *Y* and *Z* separately within each stratum. Because *X* is fixed within each subset, the imputation models within each subset of the data do not require either *X* or interactions with *X*. Thus, the imputation models become

1. [*X* fully observed; no imputation needed]
2. regression of *Z* on *Y* (within subsets of *X*)
3. regression of *Y* on *Z* (within subsets of *X*);

This approach can be modified in an obvious way when either binary *Z* or binary *Y* is the only fully observed variable.

Where the only fully observed variable is continuous, rather than categorical, which in our simulation study happens when continuous response variable *Y* is the only fully observed variable, one simple solution would be to split by the mean or median of the continuous variable. However, this may not fully account for all necessary interactions; thus, it may be desirable to additionally include the continuous variable in the imputation models for the partially observed variables.

## 3 Simulation study

We designed six sets of simulations to cover a range of possible real-life data situations, each with five different missingness mechanisms with different variables chosen to be missing, based on combinations of the other variables.

Our analysis model is the regression of *Y* on *X* and *Z* and their interaction XZ. In all six sets of simulations *X* and *Z* are binary with *X* ∼ Bernoulli(0.75), and the conditional distribution of *Z* given *X* is defined by *Z*|*X* = 0 ∼ Bernoulli(0.6), and *Z*|*X* = 1 ∼ Bernoulli(0.533). In the first three settings, *Y* is normally distributed given *X*, *Z*, and their interaction. In the remaining three settings, *Y* has a Bernoulli distribution with probabilities depending on *X*, *Z*, and their interaction. The models for the response variable are

Y=0.45X+0.55Z+ΔXZ+ε,ε∼N(0,1)

Logit{Pr(Y=1)}=0.5X+0.5Z+ΔXZ

where Δ takes the values 0.6, 0.2, and 0 in the three sets of simulations for each outcome type, representing a strong, weak, and no interaction, respectively.

For each set of data generating models mentioned previously, five different missing at random data mechanisms (A–E) were imposed (Table 1).

For each scenario (combination of six settings, and five missing data mechanisms [A–E]), we evaluate the performance of the following missing data approaches:

I. CRA
II. MI without including any interactions
III. MI including an *X*–*Z* interaction in the model used to impute the response variable *Y*, but including no interactions in other imputation models
IV. MI including all necessary interactions in all imputation models (as listed in [1]–[3] mentioned previously)
V. Stratifying by a fully observed variable. Where the only fully observed variable is continuous (mechanism D; continuous *Y* is fully observed), we created two strata by splitting *Y* at its mean and apply MI with no interactions in each stratum.

For linear regression (settings with a continuous outcome variable *Y*), we summarized the performance of each approach by the mean coefficient estimates, standard deviations of the coefficient estimates, and the estimated coverage of the 95% confidence interval for the coefficient estimate. For logistic regression (settings where *Y* is binary), we summarized the performance of each approach by the mean estimated log odds ratio (OR), the standard deviation of the estimates, and estimated coverage of the associated 95% confidence intervals.

For each scenario, the sample size was 2,000, with each scenario simulated 1,000 times. MI with 20 imputed data sets and 10 cycles of chained equations was carried out using the mi impute command in STATA (StataCorp LP) [12], [14].

In addition, to assess the behavior of these approaches in very large samples, where asymptotic statistical properties may be more apparent, we also undertook simulations with a sample size of 20,000, while increasing the number of imputed data sets to 100 and using 10 cycles of chained equations. With these more computationally intensive sample sizes, each scenario was simulated 100 times only.

## 4 Using directed acyclic graphs to establish the validity of CRA and MI

CRA leads to valid inference when the probability of a complete record, given the covariates, is unrelated to the response variable [1]. Because it is the variables causing the missing data (rather than the missing data themselves) that determine whether CRA is valid, it is natural to visualize this using a directed acyclic graph (DAG) [7], [15], [16]. A DAG is not parametric, so can be used to identify when CRA is likely to be biased irrespective of whether the analysis model includes interaction terms.

**Fig. 1:**
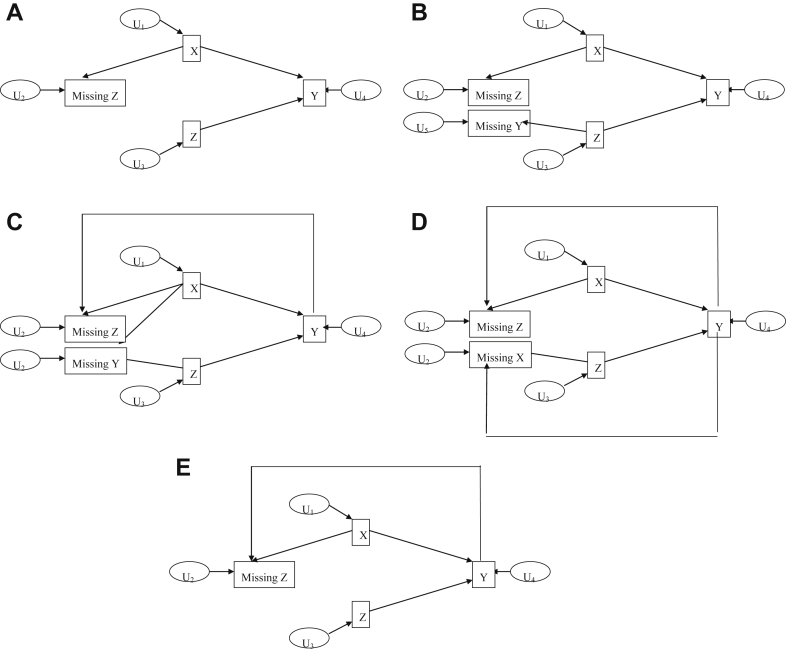
(A) DAG showing relationships between fully observed variables *X* and *Y* and partially observed variable *Z*, and missingness in *Z*, for missingness mechanism A in the simulation study. (B) DAG showing relationships between fully observed variable *X* and partially observed variables *Y* and *Z*, and missingness in *Y* and *Z*, for missingness mechanism B in the simulation study. (C) DAG showing relationships between fully observed variable *X* and partially observed variables *Y* and *Z*, and missingness in *Y* and *Z*, for missingness mechanism C in the simulation study. (D) DAG showing relationships between fully observed variable *Y* and partially observed variables *X* and *Z*, and missingness in *X* and *Z*, for missingness mechanism D in the simulation study. (E) DAG showing relationships between fully observed variables *X* and *Y* and partially observed variable *Z*, and missingness in *Z*, for missingness mechanism E in the simulation study. DAG, directed acyclic graph.

The DAGs for mechanisms A to E are shown in Fig. 1A–E, respectively. In mechanism A (Fig. 1A), conditional on *X*, the response variable *Y* is independent of the missingness mechanism. In mechanism B (Fig. 1B), conditional on *X* and *Z*, *Y* is independent of the missingness mechanism. For mechanisms C–E, the corresponding Fig. 1C–E show that *Y* is included in the missingness mechanism. From these DAGs, it is clear that CRA should be valid in mechanisms A and B only.

The DAG for each missing data mechanism can also be used to decide whether an MAR assumption is plausible (implying that MI would be valid). For this purpose, the DAG needs to identify plausible missing data mechanisms for each variable with any missing data, with the data for each variable being MAR if the missing data mechanism only depends on data that are observed. DAGs are useful here as it may be easier to construct a reasonable DAG than to write out the relevant probability distributions—they can also help in explaining these issues to collaborators, and present missing data issues alongside confounding.

Fig. 1A shows that, for mechanism A, the missingness in *Z* is independent of *Z* (conditional on *X* and *Y*), and thus, data in *Z* are MAR. In mechanism B (Fig. 1B), data in *Z* are MAR conditional on *X*, and data in *Y* are MAR conditional on *Z*. In mechanism C (Fig. 1C), data in *Z* are MAR conditional on *X* and *Y*, and data in *Y* are MAR conditional on *X* and *Y*. In mechanism D (Fig. 1D), data in *Z* are MAR conditional on *X* and *Y*, and data in *X* are MAR conditional on *Z* and *Y*. Finally, for mechanism E (Fig. 1E), data in *Z* are MAR conditional on *X* and *Y*. Thus, the DAGs reflect that for all missingness mechanisms considered here, the data are MAR, and thus a properly carried out MI (including appropriate interactions) should be valid.

## 5 Application

We have used data from the NCDS to show the application of these methods in practice [17], [18]. The NCDS is a continuing longitudinal study that follows all those living in Great Britain who were born in one particular week in 1958 [19]. Our binary outcome is “no educational qualifications,” assessed at 23 years of age. Our analysis model is a logistic regression of this outcome on maternal age at birth of child (continuous), whether the child was in social housing before age 7 (binary), and their interaction. We first carried out a complete case analysis (I mentioned previously), then MI with no interactions (II), MI correctly including the interaction terms (IV), and finally MI split on the (incomplete) social housing variable (V). We carried out 100 imputations in each case, using logistic regression to impute the outcome and the social housing exposure, and linear regression for maternal age. No other covariates were included.

## 6 Results

### 6.1 Simulation study

**Fig. 2:**
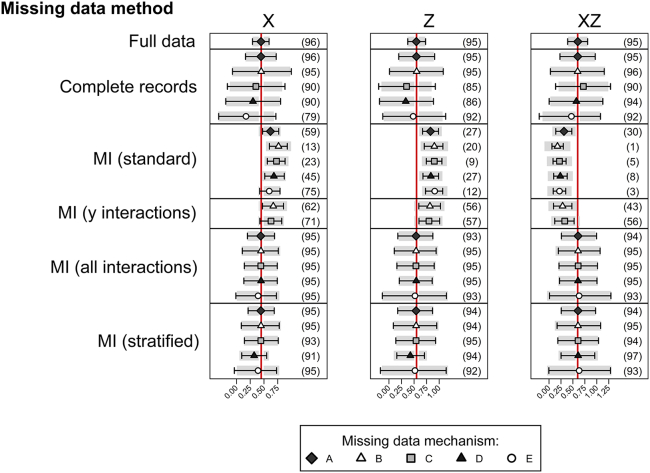
Results from 1,000 simulated data sets, each containing 2,000 individuals. The outcome is continuous, with a large *X*–*Z* interaction. The graph shows the mean (5th–95th percentiles) of the estimated coefficients across simulations with the true value indicated by the red vertical line. The estimated coverage of the 95% confidence interval is shown in parentheses. MI, multiple imputation. (For interpretation of the references to color in this figure legend, the reader is referred to the Web version of this article.)

The simulations with continuous response variable *Y* depending on binary covariates *X* and *Z* and strongly dependent on their interaction are shown in Fig. 2 (and Webtable 1/Appendix at www.jclinepi.com). In line with theory, CRA gives valid inference under mechanisms A and B, with some loss of efficiency (as shown by the larger standard deviations, compared to the full data analysis). For mechanisms C, D, and E, CRA gives invalid inference and undercoverage of confidence intervals.

MI ignoring interactions is biased with poor coverage for all parameters estimated and for all missingness mechanisms (A–E). The coverage of the confidence intervals from the multiple imputation analysis is lower than 95% in all cases, with estimates being more biased than those from CRA, particularly for the interaction term. MI only including the XZ interaction in the imputation model for *Y* performs better than MI including no interactions for mechanisms B and C (the only mechanisms where *Y* is incomplete and therefore is imputed). However, the estimates are still biased, and there is undercoverage of the confidence intervals.

As expected, MI correctly including all interactions (as described previously) gives valid inference for all missingness mechanisms A–E. There is some loss of efficiency with respect to the full data analysis but good coverage of all confidence intervals. Even where both MI and CRA were unbiased, MI is more efficient than CRA.

Finally, splitting the data gives valid inference apart from under mechanism D, where splitting the data leads to invalid inference and undercoverage. In this case, the complete variable (the response variable, *Y*) is continuous, so we split on the mean value of the observed *Y*. Thus, within each half of the data set, there is still an association between *Y* and the interaction term XZ which is not being taken account of in the simple MI in each half. Apart from this case, inferences from MI with appropriate interactions and splitting are practically identical, with slightly lower coverage rates for splitting rather than full imputation in most cases.

The simulations with continuous response variable *Y* depending weakly dependent on the XZ interaction (Webfigure 1 and Webtable 2/Appendix at www.jclinepi.com) showed similar results. The imputations with no interactions were less biased for this weak interaction than for the strong interaction, with coverage rates all above 90%.

**Fig. 3:**
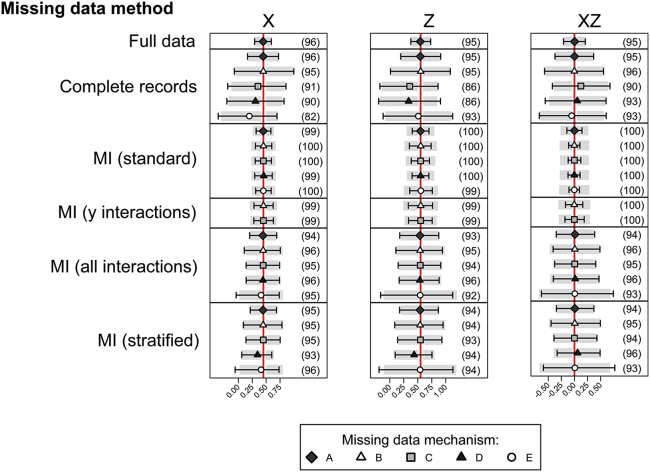
Results from 1,000 simulated data sets, each containing 2,000 individuals. The outcome is continuous, with no *X*–Z interaction. The graph shows the mean (5th–95th percentiles) of the estimated coefficients across simulations with the true value indicated by the red vertical line. The estimated coverage of the 95% confidence interval is shown in parentheses. MI, multiple imputation. (For interpretation of the references to color in this figure legend, the reader is referred to the Web version of this article.)

The simulations with continuous variable *Y* not depending on the XZ interaction are shown in Fig. 3 (Webtable 3/Appendix at www.jclinepi.com). Here, the estimates are all unbiased, apart from the stratified analysis for missingness mechanism D, as before. However, the standard MI (including no interactions) now results in noticeable overcoverage of the confidence intervals, with coverage around 99%. This is because the analyst is “assuming” less than the imputer—the analyst is allowing the possibility of an XZ interaction, whereas the imputer is (correctly) saying that the interaction is zero [20].

**Fig. 4:**
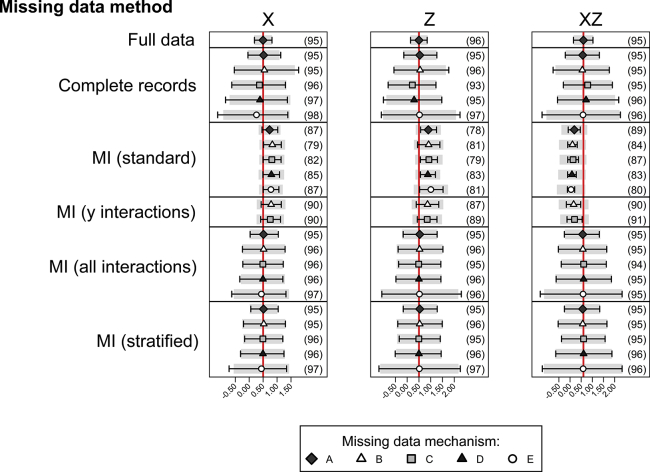
Results from 1,000 simulated data sets, each containing 2,000 individuals. The outcome is binary, with a large *X*–Z interaction. The graph shows the mean (5th–95th percentiles) of the estimated coefficients across simulations with the true value indicated by the red vertical line. The estimated coverage of the 95% confidence interval is shown in parentheses. MI, multiple imputation. (For interpretation of the references to color in this figure legend, the reader is referred to the Web version of this article.)

The simulations with binary response variable *Y* depending on binary covariates *X* and *Z* and strongly dependent on their interaction are shown in Fig. 4 (Webtable 4/Appendix at www.jclinepi.com). Here, CRA gives valid inference not only under mechanisms A and B but also for two of the parameter estimates under missingness mechanism E. In this mechanism, *Z* is partially observed dependent on *X* and *Y*, and the ORs relating *Y* to *Z* and *Y* to XZ are unbiased with good coverage. There is some loss of efficiency with CRA (with wider confidence intervals than those for the full data analysis) and MI ignoring interactions is always biased with poor coverage. MI including all interactions gives valid inference under all missingness mechanisms (A–E), with some loss of efficiency, but good coverage of all confidence intervals. Similarly, splitting the data gives valid inference under all missingness mechanisms, with inferences from MI with appropriate interactions and splitting being practically identical.

The simulations with binary response variable *Y* weakly dependent on the XZ interaction (Webfigure 2 and Webtable 5/Appendix at www.jclinepi.com) showed similar results. The imputations with no interactions were less biased for the weak interaction than for the strong interaction and the confidence intervals had coverage rates above 95%. As in the continuous case, where there is truly no XZ interaction, standard MI gives unbiased estimates but overcoverage of confidence intervals (Webfigure 3 and Webtable 6/Appendix at www.jclinepi.com).

The results for large sample sizes (*n* = 20,000) are shown in Webtables 7–12/Appendix at www.jclinepi.com. The results are similar to those discussed previously, but coverage of the MI without interactions is lower, and overcoverage where the interaction is really zero is higher.

### 6.2 Application

Data are from the 1958 NCDS, which included all births during 1 week in 1958 (*n* = 18,558) in Great Britain [19]. Of these 10,625 (57%) had complete data on the outcome and both explanatory variables. Maternal age had the highest completeness (94%), followed by social housing (78%), with the outcome being least complete (68%).

The CCA and MI estimates of associations between maternal age, social housing, their interaction, and the outcome (having no educational qualifications at age 23 years) are summarized in Table 2. The estimates did not vary widely between CRA and the different imputations, and all indicated that people with younger mothers, or who were in social housing by age 7, were more likely to have no qualifications by age 23. Both the CCA and MI correctly including interactions identified a positive interaction between maternal age and social housing, indicating no association with maternal age in children who were not in social housing. The stratified MI only included people with complete data on social housing and gave similar estimates to CCA and MI including interactions. However, using MI excluding interactions, the interaction between social housing and maternal age would have been deemed “not significant,” being biased toward the null and with a confidence interval which crossed zero.

## 7 Discussion

For valid MI, the imputation model should be at least as general as the analysis model [7], [8], [9], [10]. Interactions in the analysis model imply multiple interactions in imputation models, but this is not always understood by practitioners. In all our simulated scenarios, imputation excluding the appropriate interactions gave rise to biased estimates and undercoverage of confidence intervals. In cases with a strong XZ interaction, coverage of the imputed estimates was lower than 95%. The estimate for the interaction term was biased toward the null (as expected), and estimates for the relationship between *Y* and each of *X* and *Z* biased away from the null. Where the response variable depended weakly on an interaction term, the estimates were biased but the confidence intervals were wide and thus had greater coverage. In the applied example, the bias toward the null from the MI excluding interactions could cause this interaction to be described as “not significant” and hence lead to the erroneous conclusion that there was no interaction between maternal age and social housing in their association with later qualifications. Power to detect interactions is often low in practice, and any bias of the coefficient for an interaction toward the null can thus have a substantial impact on the conclusions drawn about differences between subgroups.

Our key finding is that applying MI without reference to any interaction structure will yield severely misleading inference when even a moderate interaction effect is present. Therefore, if there is an a priori reason for including the interaction in the analysis model, MI must take this into account. There are two ways this may be done. When one of the variables involved in the interaction is both fully observed and categorical, the data can be split into strata defined by this variable, and imputation (without interaction) carried out separately within each stratum. The second approach is not to split the data but instead include the interactions in each component of the FCS imputation models. Our results confirm that both approaches give valid inference.

Which of these two approaches should be chosen in practice? If there are no missing data in one of the variables involved in the interaction, and this variable is categorical, then splitting the data on this variable and imputing separately in the resulting strata is less computationally intensive and places no restriction on the associations within each stratum. This is particularly the case for interactions involving a multicategory variable. Furthermore, even if the categorical variable *X* identifying the strata is partially observed, provided it is appropriate to assume that the missingness mechanism for this variable does not include the response, discarding records with missing *X* and then adopting this approach will give valid, if not fully efficient, inference. However, in this setting, the alternative approach of a single imputation model with appropriate interactions has the potential to be more efficient. The latter approach is the only option for valid inference when (1) both the variables in the interaction are MAR and/or (2) the interaction involves continuous variables.

An alternative might be to impute the interaction directly, treating the interaction term as “just another variable” (the JAV approach) [21], [22]. An evaluation of statistical methods for imputation when there are missing data among the covariates in a model (i.e., the *X* and *Z* in our example) examined the performance of JAV, in which *Y*, *X*, *Z*, XZ are imputed using a joint multivariate normal model [21]. In their example, JAV performed well where *Y* was a continuous response variable and the analysis model involved an XZ interaction. However, JAV performed badly (in a different scenario) when *Y* was a binary response variable. This approach is also intuitively unappealing because the imputed interaction and main effects can be inconsistent (e.g., can have interaction = 1 but both main effects = 0).

We also illustrated the practical utility of DAGs to explore the likely effects of missingness mechanisms on general inference from CRA. The DAGs identified scenarios A and B as likely to yield unbiased inference under CRA and also showed that MI (correctly carried out) should yield valid results for all scenarios considered here. The symmetry (sometimes termed reversibility) of the OR means that in logistic regression, CRA yields valid inference for those covariates which are not involved in the missingness mechanism [20]. In practice, the DAG is likely to be more complex than in our examples, and a number of possible DAGs may all be plausible. Sensitivity analyses for the impact of missing data under the range of plausible missing data mechanisms are important [6]. The plausible mechanisms and complex relationships (e.g., interactions or nonlinearities) to be considered in the analysis model, and in all imputation models, should be informed by prior evidence and expert opinion. Where there are too many variables to make it realistic to examine every interaction or nonlinearity of interest, random forest imputation (which can include interactions and nonlinearities) may be less biased than parametric imputation [23].

The literature contains various references advising analysts of the need to structure the imputation process to be compatible with interactions in the substantive model [7], [8], [9], [10]. The effect of incompatibility is also shown when there is in truth no XZ interaction, and the multiple imputation model includes no interaction, but the analyst includes the interaction in their model. Although the interaction is “correctly” not included in the imputation model, this incompatibility between the imputation model (no interaction) and the analysis model (interaction estimated to be zero) results in overcoverage of confidence intervals [24]. Thus, if interactions are to be investigated using multiply imputed data, all interactions to be considered must be correctly included in the imputation process.

MI may be performed using joint modeling, rather than FCS. “Compatibility” between imputation and analysis models in FCS, as we have used the term here, is related to “congeniality” in relation to a well-defined Bayesian joint model as defined by Meng [24]. With categorical data, appropriate joint modeling imputation procedures that are practically equivalent to the FCS approach detailed can be naturally derived using the general location model [25], [26] or a latent normal model [27]. In the case of a mix of continuous and discrete data, where all variables may have missing observations, a general approach for congenial imputation with both nonlinear relationships and interactions is possible [28].

There are limitations to our simulation study. We only examined five missing data mechanisms here, chosen to be MAR so that MI (correctly carried out) should give unbiased results. Other missing data mechanisms may give rise to different degrees of bias—particularly depending on the strength of the interaction, as we show here. We also only examined a relatively simple set of scenarios, with only three variables in the analysis model, and no auxiliary variables in the imputation model. However, in any more complex situation, this principle will remain—the imputation models need to be compatible with the analysis model, and hence, any interactions in the analysis model must be included in all the imputation models. We could have operationalized the stratified analysis (where the complete variable was continuous) by splitting on the median rather than the mean, but it is unlikely that this would have made much difference in our situation, given that the continuous variable was normally distributed. The key point with this stratification is that no matter how it is carried out, the two groups still retain heterogeneity in the continuous variable, so the necessary interaction is not fully accounted for.

We have shown that when the analysis model includes interactions, ignoring them in MI will generally yield misleading inference. Furthermore, even when the interaction terms are in truth zero, not including them in the imputation model results in overcoverage of confidence intervals. We have set out practical, generally applicable, steps for MI which respect the interaction structure and yield valid inference.

## Figures and Tables

**Table 1:** Missingness mechanisms (A–E) applied in the simulation study

Missingness mechanism	Partially missing variables^a^	Missingness model
A	*Z*	Logit{Pr(observe Z)}=−1+2.5X
B	*Z* and *Y*	Logit{Pr(observe Z)}=−3+1.5X
		Logit{Pr(observe Y)}=−2+2.5Z
C	*Z* and *Y*	Logit{Pr(observe Z)}=−3+1.5X+Y
		Logit{Pr(observe Y)}=−2+2.5Z+2X
D	*X* and *Z*	Logit{Pr(observe X)}=−2.5+1.3Z+0.8Y
		Logit{Pr(observe Z)}=−3+1.5X+Y
E	*Z*	Logit{Pr(observe Z)}=−3+1.5X+Y

*Abbreviation:* MAR, missing at random.

a Where two variables were set to be partially missing, data were first split into two distinct halves with one variable set to be partially missing within each half of the data to preserve the MAR mechanism.

**Table 2:** Results from analysis of the NCDS

Exposure	Log OR for having no educational qualifications by age 23			
	Complete records (*n* = 10,625)	Standard MI (*n* = 18,558)	MI with interactions (*n* = 18,558)	Stratified MI (*n* = 14,550)
In social housing before age 7	0.97 (0.88, 1.06)	0.98 (0.89, 1.06)	0.98 (0.90, 1.06)	0.93 (0.84, 1.01)
Maternal age at birth of child	−0.02 (−0.03, −0.008)	−0.01 (−0.03, −0.004)	−0.02 (−0.03, −0.008)	−0.02 (−0.03, −0.008)
Interaction between maternal age and social housing	0.02 (0.003, 0.03)	0.01 (−0.004, 0.02)	0.02 (0.002, 0.03)	0.02 (0.004, 0.03)

*Abbreviations:* NCDS, National Child Development Study; OR, odds ratio; MI, multiple imputation.

Outcome variable (binary) is having no educational qualifications by age 23.

## Supplementary data

Webfigures 1–3 and Webtables 1–12
