# Supplementary material for: Appropriate inclusion of interactions was needed to avoid bias in multiple imputation
Source: J Clin Epidemiol. 2016 Dec;80:107–15. doi: 10.1016/j.jclinepi.2016.07.004 (PMC5176003; doi:10.1016/j.jclinepi.2016.07.004)
Supplement: Webfigures 1–3 and Webtables 1–12 [file mmc1.pdf]

Webfigure 1

Click here to download high resolution image

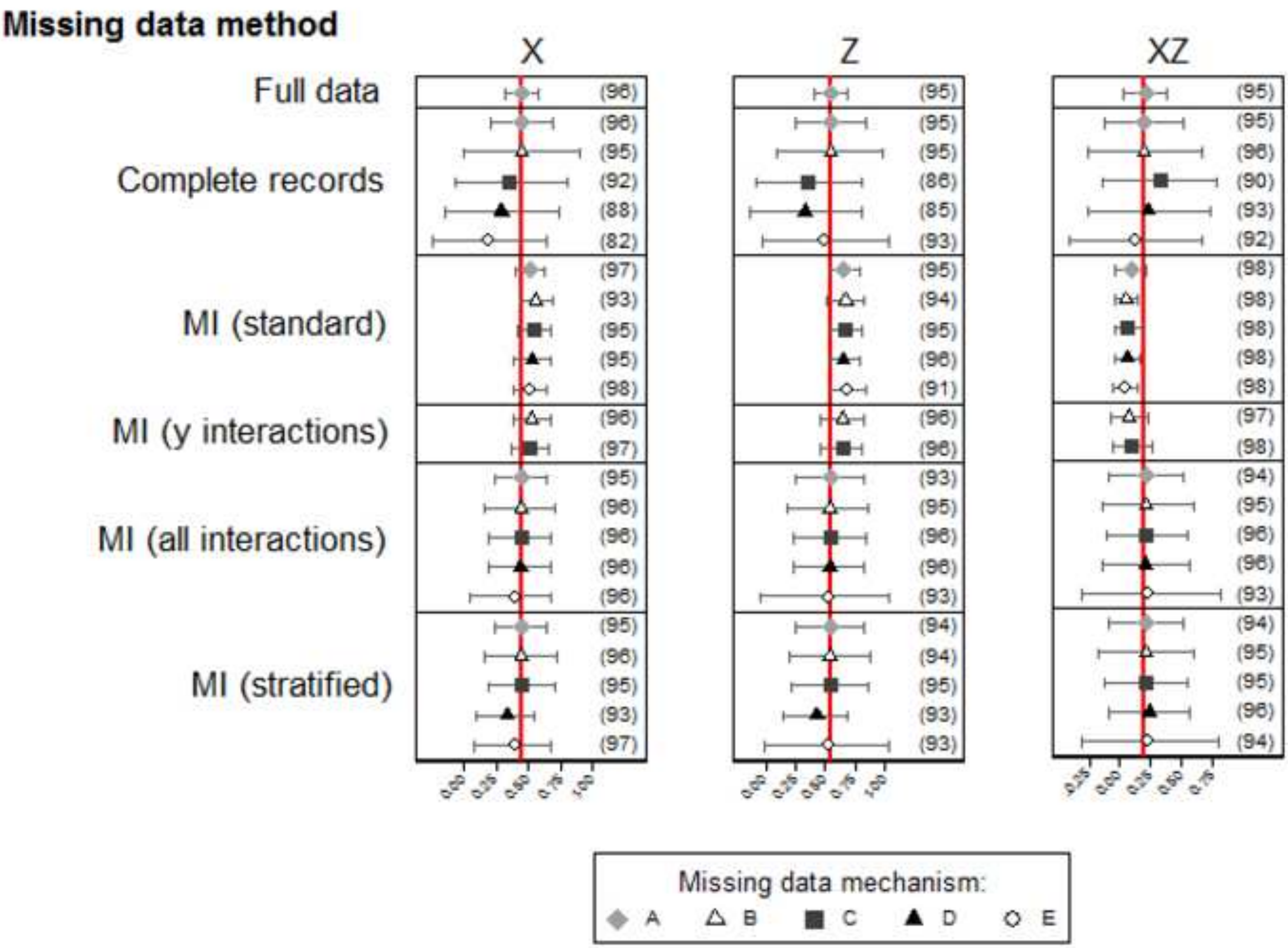

Webfigure 2

Click here to download high resolution image

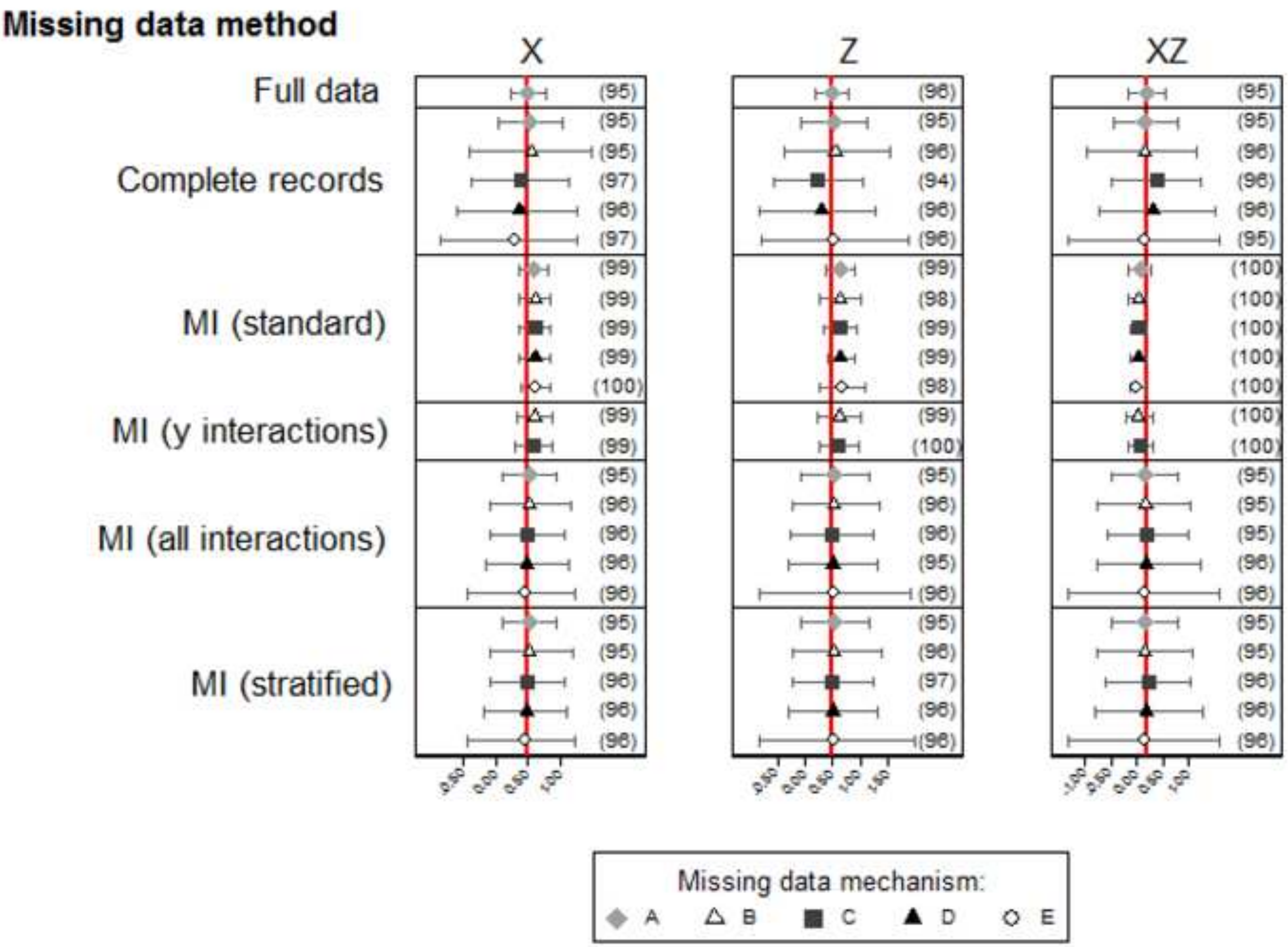

Webfigure 3

Click here to download high resolution image

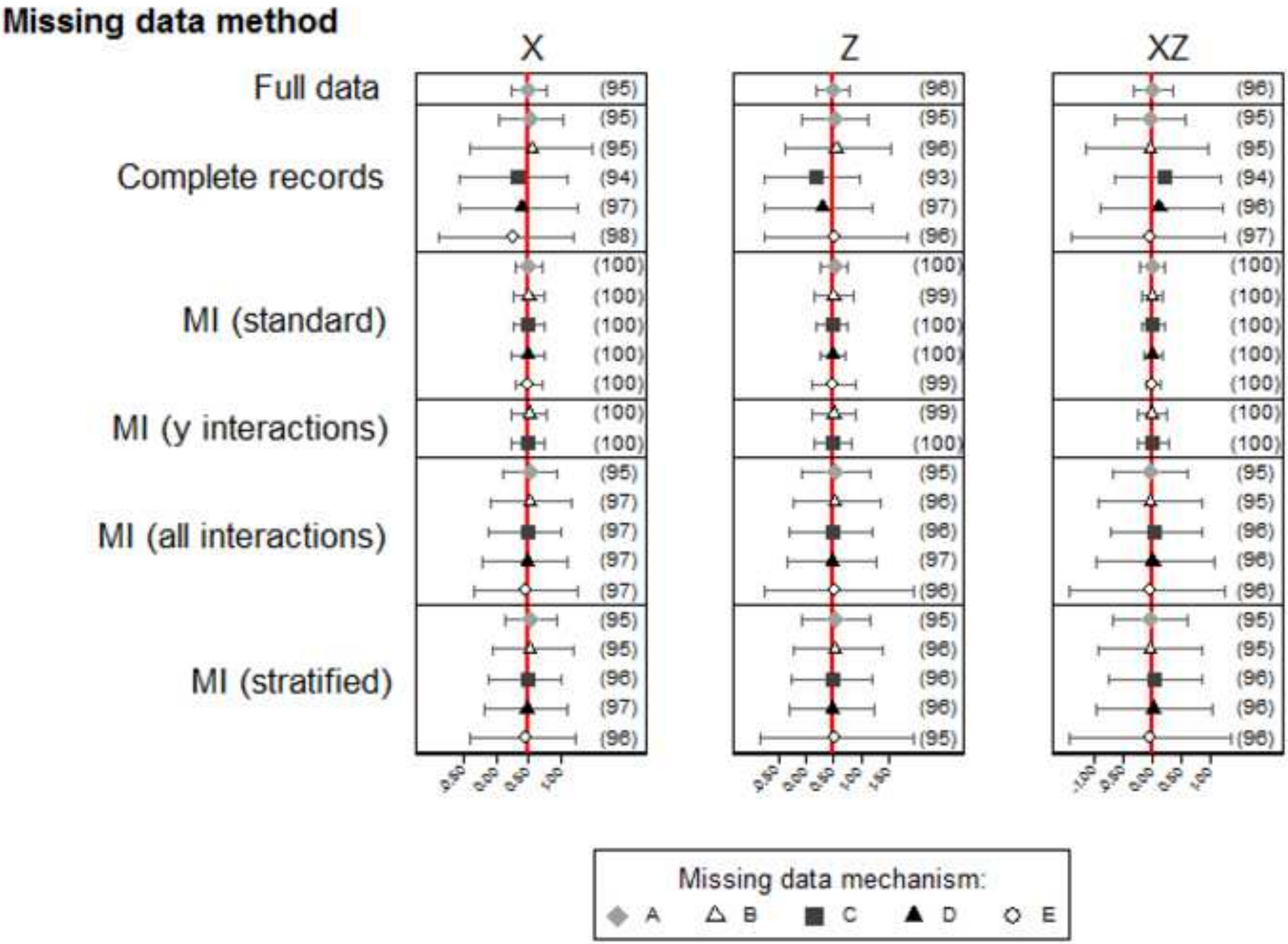

Webtable 1. Results from 1,000 simulated datasets, each containing 2,000 individuals. The outcome is **continuous**, with a **large** X-Z interaction.

		Missingness Mechanism														
		**A**			**B**			**C**			**D**			**E**		
	True value	Mean Est.	Emp. SD	Cov.	Mean Est.	Emp. SD	Cov.	Mean Est.	Emp. SD	Cov.	Mean Est.	Emp. SD	Cov.	Mean Est.	Emp. SD	Cov.
**Full data**																
	N =2,000															
X	0.45	0.447	0.079	96.0												
Z	0.55	0.546	0.090	95.1	(as for Mechanism A)			(as for Mechanism A)			(as for Mechanism A)			(as for Mechanism A)		
XZ	0.60	0.604	0.105	95.1												
**Complete records**																
	Ave. N =	*1360*			*545*			*985*			*647*			*670*		
X	0.45	0.446	0.143	95.5	0.450	0.268	95.0	0.354	0.268	90.4	0.301	0.263	89.5	0.174	0.270	78.5
Z	0.55	0.547	0.181	94.5	0.552	0.269	95.1	0.351	0.286	84.6	0.335	0.273	85.7	0.483	0.320	92.0
XZ	0.60	0.602	0.190	95.1	0.599	0.290	95.9	0.720	0.295	90.3	0.573	0.288	94.3	0.469	0.326	91.5
**Standard MI**																
X	0.45	0.619	0.076	58.7	0.765	0.081	13.0	0.728	0.083	23.4	0.680	0.091	44.6	0.597	0.094	75.0
Z	0.55	0.828	0.080	27.0	0.904	0.094	19.5	0.904	0.080	9.2	0.835	0.081	26.9	0.912	0.086	12.1
XZ	0.60	0.313	0.083	30.0	0.178	0.061	0.5	0.220	0.069	5.1	0.237	0.071	8.0	0.219	0.067	3.3
**MI with y-interaction**																
X	0.45				0.670	0.097	62.1	0.629	0.101	70.5						
Z	0.55	(as for standard MI)			0.817	0.111	55.8	0.798	0.104	57.4	(as for standard MI)			(as for standard MI)		
XZ	0.60				0.285	0.098	43.1	0.330	0.102	55.9						
**MI with interactions**																
X	0.45	0.442	0.121	94.8	0.445	0.168	95.2	0.443	0.154	95.3	0.447	0.149	94.6	0.394	0.191	94.8
Z	0.55	0.540	0.174	93.1	0.540	0.208	95.3	0.538	0.192	95.3	0.544	0.171	95.2	0.520	0.332	92.7
XZ	0.60	0.609	0.182	93.9	0.609	0.224	95.0	0.611	0.203	94.7	0.607	0.201	94.9	0.631	0.341	92.5
**Stratified MI**																
X	0.45	0.443	0.122	94.6	0.447	0.176	95.3	0.444	0.157	93.4	0.321	0.114	91.2	0.393	0.193	95.1
Z	0.55	0.541	0.175	93.8	0.541	0.216	93.8	0.541	0.197	94.8	0.427	0.140	94.3	0.518	0.334	91.6
XZ	0.60	0.608	0.183	93.9	0.607	0.235	94.5	0.609	0.209	93.8	0.611	0.179	97.2	0.632	0.344	92.5

Mean Est. = Mean of the estimated coefficients across simulations; Emp. SD = Empirical SD across simulations; Cov. = Estimated coverage of 95% confidence interval.

Webtable 2. Results from 1,000 simulated datasets, each containing 2,000 individuals. The outcome is **continuous**, with a **small** X-Z interaction.

	True value	Missingness Mechanism														
		**A**			**B**			**C**			**D**			**E**		
		Mean Est.	Emp. SD	Cov.	Mean Est.	Emp. SD	Cov.	Mean Est.	Emp. SD	Cov.	Mean Est.	Emp. SD	Cov.	Mean Est.	Emp. SD	Cov.
**Full data**																
N =2,000																
X	0.45	0.447	0.079	96.0												
Z	0.55	0.546	0.090	95.1	(as for mechanism A)			(as for mechanism A)			(as for mechanism A)			(as for mechanism A)		
XZ	0.20	0.204	0.105	95.1												
**Complete records**																
Ave. N =		*1360*			*545*			*952*			*586*			*603*		
X	0.45	0.446	0.143	95.5	0.450	0.268	95.0	0.355	0.258	91.8	0.296	0.264	88.0	0.190	0.272	81.6
Z	0.55	0.547	0.181	94.5	0.552	0.269	95.1	0.352	0.273	86.2	0.332	0.282	84.6	0.498	0.320	93.4
XZ	0.20	0.202	0.190	95.1	0.199	0.290	95.9	0.328	0.281	90.2	0.229	0.298	93.3	0.121	0.328	92.3
**Standard MI**																
X	0.45	0.514	0.071	96.8	0.559	0.076	92.6	0.549	0.074	94.7	0.533	0.085	94.5	0.513	0.079	97.7
Z	0.55	0.656	0.078	95.4	0.677	0.097	94.3	0.676	0.080	94.6	0.657	0.078	95.6	0.692	0.095	90.5
XZ	0.20	0.090	0.076	97.6	0.049	0.057	98.1	0.065	0.063	98.0	0.061	0.064	98.2	0.051	0.058	97.7
**MI with y-interaction**																
X	0.45				0.535	0.091	96.4	0.519	0.089	97.4						
Z	0.55	(as for standard MI)			0.653	0.111	95.5	0.644	0.102	96.3	(as for standard MI)			(as for standard MI)		
XZ	0.20				0.078	0.089	97.3	0.098	0.094	97.6						
**MI with interactions**																
X	0.45	0.442	0.121	94.5	0.446	0.168	95.7	0.446	0.146	96.1	0.441	0.153	95.7	0.398	0.190	95.7
Z	0.55	0.540	0.174	93.1	0.541	0.208	95.2	0.540	0.188	96.3	0.541	0.179	95.5	0.526	0.329	93.0
XZ	0.20	0.209	0.183	93.9	0.208	0.227	95.1	0.211	0.197	96.2	0.208	0.218	95.7	0.223	0.339	92.9
**Stratified MI**																
X	0.45	0.443	0.122	94.8	0.446	0.176	95.5	0.448	0.152	95.2	0.335	0.133	92.7	0.400	0.187	96.7
Z	0.55	0.541	0.175	93.7	0.541	0.216	93.8	0.544	0.196	94.5	0.434	0.163	93.0	0.529	0.326	92.5
XZ	0.20	0.208	0.184	93.9	0.207	0.237	94.8	0.207	0.206	94.9	0.237	0.204	96.4	0.220	0.336	93.9

Mean Est. = Mean of the estimated coefficients across simulations; Emp. SD = Empirical SD across simulations; Cov. = Estimated coverage of 95% confidence interval.

Webtable 3. Results from 1,000 simulated datasets, each containing 2,000 individuals. The outcome is **continuous**, with **no** X-Z interaction.

	True value	Missingness Mechanism A Mean Est.	A Emp. SD	A Cov.	B Mean Est.	B Emp. SD	B Cov.	C Mean Est.	C Emp. SD	C Cov.	D Mean Est.	D Emp. SD	D Cov.	E Mean Est.	E Emp. SD	E Cov.
**Full data**																
	N =2,000															
X	0.45	0.447	0.079	96.0												
Z	0.55	0.546	0.090	95.1	(as for mechanism A)			(as for mechanism A)			(as for mechanism A)			(as for mechanism A)		
XZ	0	0.004	0.105	95.1												
**Complete records**																
	Ave. N =	*1360*			*545*			*935*			*556*			*571*		
X	0.45	0.446	0.143	95.5	0.450	0.268	95.0	0.353	0.263	90.6	0.307	0.261	90.4	0.197	0.275	82.2
Z	0.55	0.547	0.181	94.5	0.552	0.269	95.1	0.356	0.275	85.7	0.336	0.274	86.3	0.510	0.317	92.8
XZ	0	0.002	0.190	95.1	-0.001	0.290	95.9	0.123	0.283	90.1	0.055	0.292	93.4	-0.053	0.329	93.4
**Standard MI**																
X	0.45	0.448	0.068	99.1	0.452	0.074	99.8	0.450	0.073	99.6	0.456	0.082	99.2	0.450	0.073	99.5
Z	0.55	0.548	0.077	99.9	0.552	0.099	99.5	0.550	0.084	99.9	0.550	0.075	100.0	0.555	0.101	99.1
XZ	0	-0.000	0.072	99.9	-0.002	0.055	100.0	0.000	0.062	100.0	-0.001	0.060	100.0	-0.002	0.053	100.0
**MI with y-interaction**																
X	0.45				0.453	0.088	99.0	0.451	0.090	98.5						
Z	0.55	(as for standard MI)			0.552	0.112	99.2	0.551	0.107	99.3	(as for standard MI)			(as for standard MI)		
XZ	0				-0.003	0.085	99.8	-0.001	0.093	99.6						
**MI with interactions**																
X	0.45	0.442	0.121	94.4	0.446	0.169	95.7	0.447	0.151	94.9	0.442	0.154	96.3	0.408	0.197	95.0
Z	0.55	0.540	0.174	93.2	0.541	0.208	95.2	0.544	0.196	94.2	0.537	0.184	95.7	0.544	0.333	92.3
XZ	0	0.009	0.183	94.0	0.008	0.229	95.5	0.005	0.204	95.3	0.015	0.227	96.1	0.007	0.346	92.6
**Stratified MI**																
X	0.45	0.443	0.122	94.9	0.447	0.176	95.3	0.450	0.154	94.5	0.344	0.136	93.3	0.409	0.198	96.2
Z	0.55	0.541	0.175	93.7	0.541	0.216	93.8	0.548	0.202	92.8	0.434	0.169	93.5	0.542	0.332	93.6
XZ	0	0.008	0.184	94.0	0.007	0.238	95.0	0.001	0.210	94.4	0.060	0.211	96.0	0.009	0.344	92.9

Mean Est. = Mean of the estimated coefficients across simulations; Emp. SD = Empirical SD across simulations; Cov. = Estimated coverage of 95% confidence interval.

Webtable 4. Results from 1,000 simulated datasets, each containing 2,000 individuals. The outcome is **binary**, with a **large** X-Z interaction.

		Missingness Mechanism														
		**A**			**B**			**C**			**D**			**E**		
	True value	Mean Log OR	Emp. SD	Cov.	Mean Log OR	Emp. SD	Cov.	Mean Log OR	Emp. SD	Cov.	Mean Log OR	Emp. SD	Cov.	Mean Log OR	Emp. SD	Cov.
**Full data**																
	N =2,000															
X	0.50	0.497	0.161	94.9												
Z	0.50	0.501	0.183	96.1	(as for mechanism A)			(as for mechanism A)			(as for mechanism A)			(as for mechanism A)		
XZ	0.60	0.600	0.222	94.9												
**Complete records**																
	Ave. N =	*1361*			*544*			*916*			*521*			*533*		
X	0.50	0.517	0.298	95.2	0.547	0.577	95.2	0.369	0.505	96.0	0.389	0.560	96.6	0.261	0.611	97.8
Z	0.50	0.535	0.370	95.4	0.548	0.571	96.0	0.220	0.538	93.1	0.294	0.629	95.3	0.525	0.803	96.8
XZ	0.60	0.569	0.388	95.1	0.553	0.633	95.3	0.792	0.570	95.1	0.718	0.687	95.5	0.583	0.865	95.6
**Standard MI**																
X	0.50	0.732	0.144	87.3	0.831	0.159	78.5	0.813	0.158	81.8	0.796	0.161	84.6	0.781	0.148	86.9
Z	0.50	0.898	0.173	78.0	0.911	0.238	80.9	0.923	0.189	78.7	0.877	0.162	83.2	1.007	0.257	80.9
XZ	0.60	0.193	0.140	88.8	0.109	0.110	83.5	0.136	0.122	86.6	0.086	0.099	83.2	0.047	0.084	80.1
**MI with y interaction**																
X	0.50				0.787	0.181	90.2	0.758	0.184	89.8						
Z	0.50	(as for standard MI)			0.865	0.253	86.5	0.851	0.223	88.8	(as for standard MI)			(as for standard MI)		
XZ	0.60				0.161	0.166	90.1	0.202	0.175	91.1						
**MI with interactions**																
X	0.50	0.514	0.255	94.7	0.519	0.384	95.7	0.490	0.366	95.5	0.493	0.387	96.2	0.440	0.497	96.6
Z	0.50	0.535	0.379	95.2	0.529	0.484	95.8	0.490	0.479	95.3	0.498	0.488	96.3	0.523	0.830	96.4
XZ	0.60	0.569	0.396	95.1	0.576	0.550	94.5	0.613	0.520	94.2	0.609	0.636	95.1	0.586	0.895	95.4
**Stratified MI**																
X	0.50	0.514	0.254	95.4	0.523	0.385	95.3	0.492	0.359	96.2	0.494	0.389	96.0	0.439	0.489	96.5
Z	0.50	0.535	0.379	94.9	0.532	0.486	95.9	0.494	0.468	95.4	0.498	0.487	95.6	0.519	0.820	96.7
XZ	0.60	0.569	0.395	95.0	0.570	0.553	94.6	0.608	0.509	94.9	0.608	0.638	95.7	0.588	0.884	95.7

Mean Est. = Mean of the estimated log odds ratios across simulations; Emp. SD = SD of the log odds ratios across simulations; Cov. = Estimated coverage of 95% confidence interval.

Webtable 5. Results from 1,000 simulated datasets, each containing 2,000 individuals. The outcome is **binary**, with a **small** X-Z interaction.

		Missingness Mechanism														
		**A**			**B**			**C**			**D**			**E**		
	True value	Mean Log OR	Emp. SD	Cov.	Mean Log OR	Emp. SD	Cov.	Mean Log OR	Emp. SD	Cov.	Mean Log OR	Emp. SD	Cov.	Mean Log OR	Emp. SD	Cov.
**Full data**																
	N =2,000															
X	0.50	0.497	0.161	94.9												
Z	0.50	0.501	0.183	96.1	(as for mechanism A)			(as for mechanism A)			(as for mechanism A)			(as for mechanism A)		
XZ	0.20	0.199	0.217	94.7												
**Complete records**																
	Ave. N =	*1361*			*544*			*911*			*511*			*525*		
X	0.50	0.517	0.298	95.2	0.547	0.577	95.2	0.393	0.490	97.0	0.374	0.597	95.9	0.280	0.653	97.0
Z	0.50	0.535	0.370	95.4	0.548	0.571	96.0	0.232	0.512	94.2	0.303	0.635	96.0	0.538	0.840	96.0
XZ	0.20	0.167	0.388	94.9	0.147	0.626	95.6	0.378	0.538	95.9	0.320	0.690	95.7	0.162	0.884	95.3
**Standard MI**																
X	0.50	0.581	0.136	98.6	0.613	0.148	98.6	0.610	0.150	98.9	0.605	0.157	99.1	0.606	0.132	99.5
Z	0.50	0.643	0.164	99.2	0.641	0.227	98.3	0.642	0.176	99.0	0.644	0.151	99.0	0.676	0.259	97.6
XZ	0.20	0.055	0.134	99.8	0.032	0.105	100.0	0.043	0.108	100.0	0.028	0.097	100.0	0.011	0.081	100.0
**MI with y interaction**																
X	0.50				0.604	0.172	98.7	0.589	0.169	98.6						
Z	0.50	(as for standard MI)			0.630	0.242	98.7	0.614	0.205	99.6	(as for standard MI)			(as for standard MI)		
XZ	0.20				0.043	0.158	100.0	0.069	0.158	99.9						
**MI with interactions**																
X	0.50	0.514	0.256	94.7	0.521	0.384	96.3	0.492	0.353	95.8	0.491	0.393	96.4	0.455	0.523	95.9
Z	0.50	0.535	0.379	95.0	0.528	0.484	95.8	0.485	0.457	95.6	0.511	0.488	95.4	0.541	0.859	96.1
XZ	0.20	0.167	0.396	95.1	0.168	0.542	95.4	0.213	0.487	95.3	0.204	0.622	95.7	0.159	0.902	95.9
**Stratified MI**																
X	0.50	0.514	0.254	95.2	0.526	0.384	95.2	0.491	0.352	96.2	0.489	0.395	95.9	0.456	0.520	96.2
Z	0.50	0.535	0.379	94.9	0.532	0.486	95.9	0.486	0.456	96.6	0.511	0.491	95.7	0.541	0.849	96.4
XZ	0.20	0.167	0.394	94.8	0.162	0.545	94.6	0.214	0.485	96.0	0.206	0.626	96.0	0.161	0.897	96.0

Mean Est. = Mean of the estimated log odds ratios across simulations; Emp. SD = SD of the log odds ratios across simulations; Cov. = Estimated coverage of 95% confidence interval.

Webtable 6. Results from 1,000 simulated datasets, each containing 2,000 individuals. The outcome is **binary**, with **no** X-Z interaction.

		Missingness Mechanism														
		**A**			**B**			**C**			**D**			**E**		
	True value	Mean log OR	Emp. SD	Cov.	Mean log OR	Emp. SD	Cov.	Mean Log OR	Emp. SD	Cov.	Mean Log OR	Emp. SD	Cov.	Mean Log OR	Emp. SD	Cov.
**Full data**																
	N =2,000															
X	0.50	0.497	0.161	94.9												
Z	0.50	0.501	0.183	96.1	(as for mechanism A)			(as for mechanism A)			(as for mechanism A)			(as for mechanism A)		
XZ	0	-0.002	0.214	95.6												
**Complete records**																
	Ave. N =	*1361*			*544*			*909*			*507*			*517*		
X	0.50	0.517	0.298	95.2	0.547	0.577	95.2	0.334	0.518	94.3	0.396	0.572	97.1	0.268	0.626	97.6
Z	0.50	0.535	0.370	95.4	0.548	0.571	96.0	0.190	0.525	93.0	0.288	0.601	96.6	0.527	0.787	96.0
XZ	0	-0.034	0.388	95.4	-0.055	0.624	95.3	0.213	0.557	94.3	0.112	0.659	96.4	-0.035	0.811	96.5
**Standard MI**																
X	0.50	0.502	0.133	99.6	0.504	0.145	99.9	0.492	0.142	99.7	0.497	0.155	99.6	0.502	0.129	99.5
Z	0.50	0.512	0.160	99.6	0.505	0.223	99.3	0.490	0.170	99.8	0.497	0.141	100.0	0.498	0.243	99.1
XZ	0	-0.011	0.132	100.0	-0.005	0.102	100.0	0.001	0.110	100.0	-0.001	0.093	100.0	-0.002	0.074	100.0
**MI with y interaction**																
X	0.50				0.509	0.169	99.7	0.491	0.164	99.6						
Z	0.50	(as for standard MI)			0.508	0.239	99.4	0.489	0.197	99.7	(as for standard MI)			(as for standard MI)		
XZ	0				-0.013	0.155	100.0	0.003	0.160	100.0						
**MI with interactions**																
X	0.50	0.514	0.256	95.2	0.524	0.384	96.6	0.477	0.350	96.7	0.480	0.398	96.5	0.464	0.484	96.5
Z	0.50	0.535	0.379	95.0	0.528	0.484	95.9	0.474	0.449	96.0	0.489	0.482	96.7	0.536	0.811	96.1
XZ	0	-0.034	0.396	94.9	-0.035	0.537	95.3	0.018	0.485	95.6	0.008	0.613	95.9	-0.047	0.834	96.4
**Stratified MI**																
X	0.50	0.514	0.254	95.2	0.528	0.384	95.4	0.478	0.350	96.1	0.477	0.395	96.5	0.452	0.490	95.6
Z	0.50	0.535	0.379	94.9	0.532	0.486	95.9	0.475	0.446	96.3	0.485	0.476	96.2	0.519	0.818	95.2
XZ	0	-0.034	0.394	95.1	-0.041	0.539	94.7	0.016	0.484	96.2	0.012	0.607	96.1	-0.028	0.839	96.3

Mean Est. = Mean of the estimated log odds ratios across simulations; Emp. SD = SD of the log odds ratios across simulations; Cov. = Estimated coverage of 95% confidence interval.

Webtable 7. Summary of estimates from 100 simulated datasets, each containing 20,000 individuals. The outcome is **continuous**, with a **large** X-Z interaction.

		Scenario														
		**A**			**B**			**C**			**D**			**E**		
	True value	Mean Est.	Emp. SD	Cov.	Mean Est.	Emp. SD	Cov.	Mean Est.	Emp. SD	Cov.	Mean Est.	Emp. SD	Cov.	Mean Est.	Emp. SD	Cov.
**Full data**																
N =20,000																
X	0.45	0.448	0.028	94	(as for scenario A)			(as for scenario A)			(as for scenario A)			(as for scenario A)		
Z	0.55	0.547	0.033	92												
XZ	0.60	0.604	0.036	95												
**Complete records**																
Ave. N =		*13,616*			*5453*			*9848*			*6472*			*6694*		
X	0.45	0.441	0.045	95	0.441	0.079	95	0.348	0.073	73	0.295	0.083	48	0.176	0.087	7
Z	0.55	0.536	0.055	93	0.543	0.074	98	0.339	0.083	22	0.333	0.090	26	0.492	0.104	83
XZ	0.60	0.613	0.057	94	0.611	0.085	96	0.730	0.079	64	0.573	0.095	93	0.459	0.110	67
**Standard MI**																
X	0.45	0.619	0.023	0	0.760	0.023	0	0.727	0.025	0	0.671	0.026	0	0.597	0.029	0
Z	0.55	0.824	0.025	0	0.899	0.025	0	0.897	0.028	0	0.827	0.023	0	0.908	0.023	0
XZ	0.60	0.318	0.025	0	0.184	0.018	0	0.222	0.021	0	0.241	0.021	0	0.223	0.022	0
**MI with y-interaction**																
X	0.45	(as for standard MI)			0.662	0.027	0	0.624	0.030	0	(as for standard MI)			(as for standard MI)		
Z	0.55				0.810	0.029	0	0.788	0.035	0						
XZ	0.60				0.294	0.029	0	0.335	0.031	0						
**MI with interactions**																
X	0.45	0.442	0.036	95	0.442	0.051	95	0.443	0.047	96	0.448	0.042	96	0.445	0.059	96
Z	0.55	0.536	0.051	95	0.542	0.059	96	0.536	0.064	95	0.546	0.049	95	0.548	0.108	91
XZ	0.60	0.614	0.053	94	0.610	0.068	94	0.610	0.064	95	0.605	0.061	95	0.601	0.116	92
**Stratified MI**																
X	0.45	0.442	0.036	96	0.441	0.052	96	0.444	0.048	96	0.325	0.034	10	0.445	0.059	95
Z	0.55	0.536	0.050	95	0.541	0.059	98	0.537	0.066	94	0.428	0.046	33	0.548	0.107	90
XZ	0.60	0.613	0.052	94	0.611	0.069	95	0.609	0.066	96	0.608	0.059	97	0.601	0.115	92

Mean Est. = Mean of the estimated coefficients across simulations; Emp. SD = Empirical SD across simulations; Cov. = Estimated coverage of 95% confidence interval.

Webtable 8. Summary of estimates from 100 simulated datasets, each containing 20,000 individuals. The outcome is **continuous**, with a **small** X-Z interaction.

		Scenario														
		**A**			**B**			**C**			**D**			**E**		
	True value	Mean Est.	Emp. SD	Cov.	Mean Est.	Emp. SD	Cov.	Mean Est.	Emp. SD	Cov.	Mean Est.	Emp. SD	Cov.	Mean Est.	Emp. SD	Cov.
**Full data**																
N =20,000																
X	0.45	0.448	0.028	94												
Z	0.55	0.547	0.033	92	(as for scenario A)			(as for scenario A)			(as for scenario A)			(as for scenario A)		
XZ	0.20	0.204	0.036	95												
**Complete records**																
Ave. N =		*13,616*			*5453*			*9506*			*5852*			*6036*		
X	0.45	0.441	0.045	95	0.441	0.079	95	0.349	0.080	75	0.288	0.075	47	0.186	0.082	7
Z	0.55	0.536	0.055	93	0.543	0.074	98	0.352	0.079	28	0.331	0.081	23	0.499	0.092	91
XZ	0.20	0.213	0.057	94	0.211	0.085	96	0.330	0.084	64	0.241	0.087	92	0.119	0.098	84
**Standard MI**																
X	0.45	0.514	0.022	38	0.555	0.022	4	0.547	0.024	11	0.531	0.025	29	0.513	0.027	46
Z	0.55	0.652	0.025	17	0.674	0.025	6	0.678	0.025	4	0.655	0.024	10	0.688	0.027	3
XZ	0.20	0.093	0.022	12	0.054	0.016	0	0.064	0.020	0	0.065	0.021	1	0.052	0.018	0
**MI with y-interaction**																
X	0.45				0.527	0.026	55	0.519	0.030	58						
Z	0.55	(as for standard MI)			0.649	0.029	43	0.646	0.031	38	(as for standard MI)			(as for standard MI)		
XZ	0.20				0.085	0.026	22	0.097	0.030	35						
**MI with interactions**																
X	0.45	0.442	0.036	94	0.441	0.052	94	0.451	0.047	98	0.442	0.045	98	0.448	0.054	96
Z	0.55	0.536	0.051	95	0.542	0.059	96	0.551	0.057	99	0.539	0.053	94	0.556	0.093	95
XZ	0.20	0.213	0.053	94	0.212	0.069	94	0.201	0.061	96	0.215	0.067	93	0.191	0.100	95
**Stratified MI**																
X	0.45	0.442	0.036	96	0.440	0.053	95	0.451	0.047	97	0.335	0.036	24	0.448	0.054	95
Z	0.55	0.536	0.050	95	0.541	0.059	98	0.551	0.058	98	0.430	0.046	40	0.556	0.094	95
XZ	0.20	0.213	0.052	94	0.213	0.070	93	0.201	0.061	95	0.247	0.058	94	0.191	0.101	96

Mean Est. = Mean of the estimated coefficients across simulations; Emp. SD = Empirical SD across simulations; Cov. = Estimated coverage of 95% confidence interval.

Webtable 9. Summary of estimates from 100 simulated datasets, each containing 20,000 simulated individuals. The outcome is **continuous**, with **no** X-Z interaction.

	True value	Scenario A Mean Est.	A Emp. SD	A Cov.	B Mean Est.	B Emp. SD	B Cov.	C Mean Est.	C Emp. SD	C Cov.	D Mean Est.	D Emp. SD	D Cov.	E Mean Est.	E Emp. SD	E Cov.
**Full data**																
N =20,000																
X	0.45	0.448	0.028	94	(as for scenario A)			(as for scenario A)			(as for scenario A)			(as for scenario A)		
Z	0.55	0.547	0.033	92												
XZ	0	0.004	0.036	95												
**Complete records**																
Ave. N =		*13,616*			*5453*			*9355*			*5562*			*5729*		
X	0.45	0.441	0.045	95	0.441	0.079	95	0.353	0.086	70	0.304	0.081	48	0.181	0.071	7
Z	0.55	0.536	0.055	93	0.543	0.074	98	0.353	0.092	36	0.342	0.085	29	0.484	0.094	88
XZ	0	0.013	0.057	94	0.011	0.085	96	0.127	0.095	68	0.054	0.088	93	-0.028	0.098	94
**Standard MI**																
X	0.45	0.448	0.021	100	0.448	0.022	100	0.453	0.023	100	0.449	0.023	100	0.451	0.021	100
Z	0.55	0.545	0.025	100	0.551	0.026	100	0.551	0.027	100	0.552	0.021	99	0.550	0.030	100
XZ	0	0.004	0.021	100	0.002	0.015	100	-0.001	0.019	100	-0.000	0.015	100	0.001	0.017	100
**MI with y-interaction**																
X	0.45	(as for standard MI)			0.446	0.025	100	0.453	0.029	98	(as for standard MI)			(as for standard MI)		
Z	0.55				0.549	0.029	100	0.552	0.033	100						
XZ	0				0.004	0.025	100	-0.002	0.029	100						
**MI with interactions**																
X	0.45	0.442	0.036	94	0.440	0.052	94	0.453	0.049	94	0.448	0.044	97	0.439	0.050	96
Z	0.55	0.536	0.051	95	0.542	0.059	97	0.552	0.064	92	0.551	0.052	99	0.535	0.092	96
XZ	0	0.013	0.053	94	0.012	0.070	95	-0.002	0.067	94	0.000	0.063	97	0.017	0.098	96
**Stratified MI**																
X	0.45	0.442	0.036	96	0.439	0.053	94	0.453	0.050	95	0.346	0.044	32	0.439	0.050	97
Z	0.55	0.536	0.050	95	0.541	0.059	98	0.551	0.065	96	0.438	0.053	49	0.535	0.093	97
XZ	0	0.013	0.052	94	0.014	0.071	93	-0.002	0.068	96	0.059	0.066	88	0.017	0.099	98

Mean Est. = Mean of the estimated coefficients across simulations; Emp. SD = Empirical SD across simulations; Cov. = Estimated coverage of 95% confidence interval.

Webtable 10. Summary of estimated log odds ratios from 100 simulated datasets, each containing 20,000 individuals. The outcome is **binary**, with a **large** X-Z interaction.

		Scenario														
			**A**			**B**			**C**			**D**			**E**	
	True value	Mean Est.	Emp. SD	Cov.	Mean Est.	Emp. SD	Cov.	Mean Est.	Emp. SD	Cov.	Mean Est.	Emp. SD	Cov.	Mean Est.	Emp. SD	Cov.
**Full data**																
N =20,000																
X	0.50	0.506	0.051	94												
Z	0.50	0.505	0.063	92		(as for scenario A)			(as for scenario A)			(as for scenario A)			(as for scenario A)	
XZ	0.60	0.597	0.072	91												
**Complete records**																
Ave. N =		*13,609*			*5453*			*9165*			*5212*			*5315*		
X	0.50	0.510	0.088	96	0.520	0.171	95	0.392	0.164	86	0.408	0.176	89	0.321	0.215	79
Z	0.50	0.506	0.108	95	0.508	0.186	93	0.243	0.160	64	0.321	0.176	85	0.547	0.242	93
XZ	0.60	0.597	0.111	97	0.581	0.206	96	0.768	0.175	84	0.696	0.209	88	0.551	0.271	92
**Standard MI**																
X	0.50	0.731	0.039	0	0.826	0.047	0	0.809	0.044	0	0.792	0.055	0	0.786	0.049	0
Z	0.50	0.886	0.051	0	0.893	0.073	0	0.918	0.053	0	0.876	0.049	0	1.001	0.070	0
XZ	0.60	0.203	0.039	0	0.111	0.034	0	0.137	0.034	0	0.088	0.028	0	0.045	0.023	0
**MI with y-interaction**																
X	0.50				0.776	0.054	0	0.752	0.050	3						
Z	0.50		(as for standard MI)		0.843	0.077	3	0.848	0.062	1		(as for standard MI)			(as for standard MI)	
XZ	0.60				0.172	0.053	0	0.204	0.050	0						
**MI with interactions**																
X	0.50	0.505	0.071	97	0.507	0.123	94	0.496	0.100	98	0.509	0.119	95	0.525	0.161	89
Z	0.50	0.506	0.108	94	0.501	0.159	93	0.496	0.134	97	0.509	0.142	94	0.545	0.244	92
XZ	0.60	0.597	0.112	97	0.590	0.181	94	0.604	0.147	94	0.596	0.191	95	0.554	0.274	91
**Stratified MI**																
X	0.50	0.504	0.070	97	0.506	0.126	94	0.498	0.101	98	0.510	0.119	96	0.523	0.159	90
Z	0.50	0.505	0.107	95	0.501	0.163	94	0.499	0.135	95	0.510	0.143	95	0.542	0.244	92
XZ	0.60	0.597	0.111	97	0.591	0.183	91	0.601	0.147	93	0.596	0.192	93	0.556	0.272	92

Mean Est. = Mean of the estimated coefficients across simulations; Emp. SD = Empirical SD across simulations; Cov. = Estimated coverage of 95% confidence interval.

Webtable 11. Summary of estimated log odds ratios from 100 simulated datasets, each containing 20,000 individuals. The outcome is **binary**, with a **small** X-Z interaction.

	True value	Scenario A Mean Est.	A Emp. SD	A Cov.	B Mean Est.	B Emp. SD	B Cov.	C Mean Est.	C Emp. SD	C Cov.	D Mean Est.	D Emp. SD	D Cov.	E Mean Est.	E Emp. SD	E Cov.
**Full data**																
N =20,000																
X	0.50	0.506	0.051	94	(as for scenario A)			(as for scenario A)			(as for scenario A)			(as for scenario A)		
Z	0.50	0.505	0.063	92												
XZ	0.20	0.198	0.069	93												
**Complete records**																
Ave. N =		*13,609*			*5453*			*9111*			*5127*			*5223*		
X	0.50	0.510	0.088	96	0.520	0.171	95	0.371	0.137	91	0.431	0.171	93	0.321	0.163	89
Z	0.50	0.506	0.108	95	0.508	0.186	93	0.232	0.144	60	0.331	0.181	84	0.524	0.203	99
XZ	0.20	0.198	0.111	96	0.182	0.201	95	0.379	0.154	85	0.282	0.200	90	0.174	0.223	98
**Standard MI**																
X	0.50	0.582	0.038	86	0.611	0.044	77	0.602	0.045	73	0.598	0.047	81	0.597	0.036	78
Z	0.50	0.634	0.049	70	0.630	0.072	77	0.643	0.051	68	0.625	0.047	73	0.669	0.080	72
XZ	0.20	0.065	0.037	84	0.035	0.032	70	0.043	0.030	77	0.029	0.025	71	0.014	0.018	57
**MI with y-interaction**																
X	0.50	(as for standard MI)			0.595	0.051	89	0.581	0.051	90	(as for standard MI)			(as for standard MI)		
Z	0.50				0.614	0.076	87	0.067	0.044	93						
XZ	0.20				0.055	0.050	83	0.067	0.044	93						
**MI with interactions**																
X	0.50	0.505	0.071	97	0.506	0.121	93	0.496	0.098	97	0.495	0.112	96	0.508	0.127	97
Z	0.50	0.506	0.108	94	0.501	0.159	93	0.502	0.125	97	0.494	0.140	97	0.526	0.204	98
XZ	0.20	0.197	0.112	96	0.191	0.176	93	0.198	0.135	95	0.205	0.177	96	0.171	0.225	97
**Stratified MI**																
X	0.50	0.505	0.070	96	0.505	0.124	91	0.496	0.096	98	0.496	0.111	97	0.504	0.124	98
Z	0.50	0.505	0.107	95	0.501	0.163	94	0.501	0.122	98	0.495	0.139	98	0.519	0.200	99
XZ	0.20	0.198	0.110	97	0.192	0.178	93	0.200	0.132	96	0.204	0.176	98	0.178	0.221	99

Mean Est. = Mean of the estimated coefficients across simulations; Emp. SD = Empirical SD across simulations; Cov. = Estimated coverage of 95% confidence interval.

Webtable 12. Summary of estimated log odds ratios from 100 simulated datasets, each containing 20,000 individuals. The outcome is **binary**, with **no** X-Z interaction.

		Scenario														
		**A**			**B**			**C**			**D**			**E**		
	True value	Mean Est.	Emp. SD	Cov.	Mean Est.	Emp. SD	Cov.	Mean Est.	Emp. SD	Cov.	Mean Est.	Emp. SD	Cov.	Mean Est.	Emp. SD	Cov.
**Full data**																
	N =20,000															
X	0.50	0.506	0.051	94	(as for scenario A)			(as for scenario A)			(as for scenario A)			(as for scenario A)		
Z	0.50	0.505	0.063	92												
XZ	0	-0.003	0.068	94												
**Complete records**																
	Ave. N =	*13,609*			*5453*			*9073*			*5058*			*5169*		
X	0.50	0.510	0.088	96	0.520	0.171	95	0.369	0.154	85	0.426	0.176	93	0.298	0.186	80
Z	0.50	0.506	0.108	95	0.508	0.186	93	0.215	0.168	54	0.316	0.190	83	0.501	0.237	97
XZ	0	-0.004	0.110	94	-0.017	0.201	96	0.195	0.175	77	0.063	0.198	91	-0.006	0.248	98
**Standard MI**																
X	0.50	0.504	0.037	100	0.501	0.043	100	0.495	0.046	100	0.496	0.053	100	0.494	0.042	100
Z	0.50	0.503	0.048	100	0.497	0.069	100	0.494	0.054	100	0.498	0.046	100	0.495	0.079	99
XZ	0	-0.002	0.037	100	-0.001	0.032	100	0.005	0.034	100	-0.005	0.026	100	0.001	0.020	100
**MI with y-interaction**																
X	0.50	(as for standard MI)			0.501	0.050	100	0.492	0.055	100	(as for standard MI)			(as for standard MI)		
Z	0.50				0.496	0.073	99	0.490	0.065	100						
XZ	0				-0.001	0.049	100	0.008	0.053	100						
**MI with interactions**																
X	0.50	0.506	0.071	97	0.505	0.121	95	0.482	0.112	95	0.512	0.121	96	0.493	0.147	95
Z	0.50	0.506	0.108	94	0.501	0.159	93	0.476	0.148	93	0.519	0.138	95	0.503	0.237	96
XZ	0	-0.005	0.111	95	-0.006	0.175	93	0.023	0.157	98	-0.035	0.178	97	-0.006	0.249	97
**Stratified MI**																
X	0.50	0.505	0.070	96	0.505	0.124	93	0.480	0.113	92	0.514	0.123	94	0.491	0.147	96
Z	0.50	0.505	0.107	95	0.501	0.163	94	0.475	0.149	92	0.522	0.142	93	0.499	0.239	95
XZ	0	-0.004	0.110	96	-0.006	0.178	92	0.025	0.157	97	-0.038	0.181	95	-0.004	0.249	99

Mean Est. = Mean of the estimated coefficients across simulations; Emp. SD = Empirical SD across simulations; Cov. = Estimated coverage of 95% confidence interval.

WebFigure 1: Results from 1,000 simulated datasets, each containing 2,000 individuals. The outcome is **continuous**, with a **small** X-Z interaction. The graph shows the mean (5th-95th percentiles) of the estimated coefficients across simulations with the true value indicated by the red vertical line. The estimated coverage of the 95% confidence interval is shown in parentheses.

Webfigure 2: Results from 1,000 simulated datasets, each containing 2,000 individuals. The outcome is **binary**, with a **small** X-Z interaction. The graph shows the mean (5th-95th percentiles) of the estimated coefficients across simulations with the true value indicated by the red vertical line. The estimated coverage of the 95% confidence interval is shown in parentheses.

Webfigure 3: Results from 1,000 simulated datasets, each containing 2,000 individuals. The outcome is **binary**, with **no** X-Z interaction. The graph shows the mean (5th-95th percentiles) of the estimated coefficients across simulations with the true value indicated by the red vertical line. The estimated coverage of the 95% confidence interval is shown in parentheses.
